# Single-cell profiling to transform immunotherapy usage and target discovery in immune-mediated inflammatory diseases

**DOI:** 10.3389/fimmu.2022.1006944

**Published:** 2022-11-07

**Authors:** Nicolas Chapelle, Aurelie Fantou, Thomas Marron, Ephraim Kenigsberg, Miriam Merad, Jerome C. Martin

**Affiliations:** ^1^ Nantes Université, CHU Nantes, INSERM, Center for Research in Transplantation and Translational Immunology, Nantes, France; ^2^ CHU Nantes, Nantes Université, Laboratoire d’Immunologie, Nantes, France; ^3^ Precision Immunology Institute, Icahn School of Medicine at Mount Sinai, New York, NY, United States; ^4^ Tisch Cancer Institute, Icahn School of Medicine at Mount Sinai, New York, NY, United States; ^5^ Department of Oncological Sciences, Icahn School of Medicine at Mount Sinai, New York, NY, United States; ^6^ Human Immune Monitoring Center, Icahn School of Medicine at Mount Sinai, New York, NY, United States

**Keywords:** human disease pathophysiology, single-cell technologies, drug discovery, immunotherapy, clinical translation, immune-mediated inflammatory diseases

## Abstract

Immunotherapy drugs are transforming the clinical care landscape of major human diseases from cancer, to inflammatory diseases, cardiovascular diseases, neurodegenerative diseases and even aging. In polygenic immune-mediated inflammatory diseases (IMIDs), the clinical benefits of immunotherapy have nevertheless remained limited to a subset of patients. Yet the identification of new actionable molecular candidates has remained challenging, and the use of standard of care imaging and/or histological diagnostic assays has failed to stratify potential responders from non-responders to biotherapies already available. We argue that these limitations partly stem from a poor understanding of disease pathophysiology and insufficient characterization of the roles assumed by candidate targets during disease initiation, progression and treatment. By transforming the resolution and scale of tissue cell mapping, high-resolution profiling strategies offer unprecedented opportunities to the understanding of immunopathogenic events in human IMID lesions. Here we discuss the potential for single-cell technologies to reveal relevant pathogenic cellular programs in IMIDs and to enhance patient stratification to guide biotherapy eligibility and clinical trial design.

## Introduction

Pioneered by anti–tumor necrosis factor (TNF) antibodies (Abs), the introduction of targeted therapies against inflammatory cytokines transformed the clinical outcome of patients with major chronic immune-mediated inflammatory diseases (IMIDs), such as rheumatoid arthritis (RA), psoriasis and inflammatory bowel disease (IBD) (1–3). More recently, the blockade of immune checkpoints resulted in a major paradigm shift in cancer care, which was acknowledged by the 2018 Nobel prize awards in physiology or medicine (4). These spectacular achievements emphasize the extraordinary potential for the discovery of new immunotherapy drug targets that can be leveraged from the vast immunology knowledge generated over the past century.

In monogenic forms of IMIDs where disease pathophysiology is predominantly driven by a unique molecular pathway, substantial clinical success has been achieved. This is for instance the case in autoinflammatory diseases driven by sustained activation of the inflammasome, such as familial Mediterranean fever (FMF) or cryopyrin-associated periodic syndrome (CAPS) (5, 6). Substantial clinical success was also achieved in cancer lesions expressing mismatch repair insufficiency, which drives the expression of high number of neoantigens and increases the immunogenicity of tumors and their responsiveness to PD-1 blockade. This led for the first time in the history of cancer to the FDA approval of PD-1 blockade in all microsatellite instability-high (MSI-H) cancers, regardless of tumor types (7–9).

In contrast, the clinical benefits of immunotherapy in polygenic IMIDs have remained limited to a subset of patients. About 30-40% of IBD patients do not respond or end up not responding to anti-TNF Abs (10, 11). Only two targeted therapies with overall moderate benefits, anifrolumab and belimumab, have been approved for the management of systemic lupus erythematosus (SLE) in more than 50 years of research (12, 13). The limited benefits of immunotherapy in IMIDs partly account for the immunopathological heterogeneity present across similar tissue lesions in IMIDs, as long-time supported by the pleiomorphic auto-antibody patterns for instance. However, while it is now clear that clinical and histological diagnoses are not sufficient to predict efficacy, none of the targeted immunotherapies approved to treat polygenic IMIDs are provided with decision algorithms to maximize chances of therapeutic response.

### Genetics of diseases dominate pathophysiology studies

The sequencing revolution that has occurred over the last decades has propelled enormous enthusiasm and surge to identify the underlying genetic drivers of diseases. Genetic profiling has led to significant advances in the identification, understanding and treatment of monogenic immune diseases such as primary immunodeficiencies (severe combined immunodeficiency (SCID), common variable immunodeficiency (CVID) etc…), auto-inflammatory diseases (FMF, tumor necrosis factor receptor associated periodic syndrome (TRAPS), CAPS, Blau syndrome etc…) and monogenic autoimmune diseases (autoimmune polyendocrinopathy candidiasis ectodermal dystrophy (APECED), immunodysregulation polyendocrinopathy enteropathy X-linked (IPEX), (cytotoxic T-lymphocyte-associated protein-4) CTLA4 deficiency etc…) (5, 14–16). In rare cases of exceptionally strong gene linkage, genetic testing can even contribute to strengthening the diagnosis of multifactorial polygenic diseases such as HLA-B27 in ankylosing spondylitis or HLA-DQ2/DQ8 in celiac disease.

While the clinical benefits of genetic testing are limited to individuals at risk, genome-wide association studies (GWAS) have been extensively used to identify potential mechanisms of diseases in larger patient populations. In most IMIDs, however, only a minor fraction of disease trait variability has been linked to specific genomic loci, and risk gene variants confer small and unpredictable risk of disease occurrence. The role for extragenetic factors has been supported by epidemiologic observations showing discordance of disease expression between homozygous twins, onset frequently occurring during adulthood (17), and the “outbreak” of IMIDs upon change of lifestyle and dietary habits. Accordingly, environmental exposure, infectious history, microbiome composition and epigenetic factors are considered as significant contributors to disease onset, evolution and response to treatment (18, 19). It is thus unlikely that genetic profiling alone will ever be sufficient to help diagnose or predict response to immunotherapy drugs in polygenic IMIDs (17, 20).

### Cellular and tissue-specific gene defects drive disease pathophysiology

Genetic studies should by no means be reduced but it is urgent that we enhance our efforts to understand the cellular contribution to disease pathophysiology. Functional genetics can map risk loci or genes to a cellular phenotype but cellular- and tissue-specific effects of gene mutations often lead to distinct disease pathophysiology. This has been well demonstrated in cancer lesions, where loss-of-function or gain-of-function alterations in specific genes can promote tumorigenesis in some tissues while ineffectual in others (21). For example, inherited BRCA1- and BRCA2-inactivating mutations predispose mainly to breast and ovarian cancer; adenomatous polyposis coli (APC) to colorectal cancer (CRC); and KRAS mutations to pancreas, colon and lung cancer, emphasizing the contribution of the tissue environment to the gene effect (21). IMIDs sharing genetic susceptibility within the IL-23/IL-17 pathway respond differently to IL-17 blockade, and while significant clinical benefit was obtained in axial spondyloarthritis (22) and psoriasis (23, 24), IL-17 blockade led to disease worsening in ileal Crohn’s disease (25). Thus, the pathogenicity of a specific pathway and the outcome of its blockade in polygenic IMIDs may vary across cell types, tissue types but also biological context (26).

Immunologists have excelled in cellular taxonomy (i.e., the definition of cellular phenotypic identity and functional specialization) but the nature of the cellular and molecular pathways that lead to human immune diseases remain sparse. Improving the prediction on gene effects and their therapeutic potential will necessarily depend on the understanding of the cellular phenotypes that populate the diseased organs. In that regard, recent data have emerged to support the potential of population-scale single-cell RNA sequencing (scRNA-seq) analyses to explore multiple expression quantitative trait locus (eQTL) in a cell type-specific manner (26, 27). Combined with the need to gain a better understanding of the cellular programs shaping normal and lesional tissues, such approaches could be instrumental for our understanding of causal mechanisms of complex diseases.

Before that, however, efforts to improve the access to biological samples and the even rarer access to longitudinal sampling of human lesions during disease progression or during treatment must be initiated, as discussed below.

### The struggle to discover new drug targets and the need to facilitate human tissue access to scientists

Most drug targets fail to succeed when evaluated in phase 3 trials, hence accounting for both an important economic burden and a distraction for physicians and scientists, which adversely impacts clinical care. The reasons leading to failure are numerous and include factors such as safety issues, insufficient enrollment, suboptimal study design and chiefly, the inability to demonstrate drug efficacy (28). In fact, the majority of FDA-approved drugs every year target known pathophysiological pathways, reflecting the struggle to identify new actionable molecular candidates. We argue that these limitations partly stem from a poor understanding of disease pathophysiology and insufficient characterization of the roles assumed by candidate targets during disease initiation, progression or response to treatment. Animal models led to many key discoveries in immunology, including in the biomedical field (29) but discrepancies between animal and human pathophysiology have been raised as possible obstacles. Still, experiments in mice enabled the development of anti-TNF therapy for RA (30), and of Abs targeting immune checkpoints in cancer (31). While we should discard models that have consistently proved unable to represent human disease, we believe that the limited cellular and molecular information about human lesions actually precluded the development of appropriate models for preclinical studies.

High dimensional mapping strategies now provide with unique opportunities to thoroughly describe the sequence of immunopathogenic events developing along disease course and therapeutic intervention in human lesions (32). Ambitious programs aiming to build data-derived hypotheses as main drivers of mechanistic explorations nevertheless come with heavy loads of administrative and organizational work for both clinical and scientific teams. For immunologists, there is an ever urgent need to access freshly collected tissues as the functionality of immune cells can only be fully revealed on perturbed live cells. It appears critical that such programs be fully embraced at the institutional level to succeed. For instance, by investing more efforts into the creation of professional teams dedicated to patient selection, consent and inclusion, as well as clinical data and tissue collection, distribution, storage and retrieval, the collaboration between physicians and scientists could be largely facilitated. It will thus be important that clinical and scientific teams work in close collaboration together with institutional but also ethical committees to the design of studies offering guarantees of sufficient sustainability (33).

Longitudinal profiling studies, in particular, require that ethically-acceptable conditions be defined and properly framed by ethical review boards. While repeated sampling in organs that would put the patient at risk with no direct clinical needs is simply not ethically acceptable, it remains possible in tissues such as the skin or the digestive tract. In addition, because histology-based decisions are gaining prominence in the therapeutic management of patients with IBD or chronic inflammatory lesions of the stomach for instance (34, 35), this makes it even possible to align molecular profiling with clinical care. IMIDs share several targetable molecular drivers (36, 37), and one can anticipate diseases involving organs not easily accessible to biopsy collection will still benefit from knowledge gains achieved through such longitudinal studies.

### High dimensional single-cell technologies: enhancing immunopathology knowledge in IMIDs for drug target discovery and patient stratification

Empowered by the progress of next generation sequencing (NGS) technologies, genomic assays are currently being used to search for molecular patterns that can stratify patients’ lesions beyond standard histological assays. Genomic assays that include transcriptomic information contributed to the identification of several molecular signatures of IMIDs, some of which have been successfully correlated to distinct clinical outcomes (38–48). Strategies profiling tissues in bulk only inform on the average expression profile of a mixture of different cell types and cannot resolve cell-specific gene expression programs (49). The development of high-resolution profiling approaches including single-cell technologies have significantly enhanced information obtained from limited human biological specimens. The use of massively parallel single-cell genomics assays can now routinely profile the transcriptome of tens of thousands of cells. Beyond RNA, it is possible to measure DNA, protein and epigenomic profiles with single cell resolution (32). Spatial mapping technologies, which inform on cellular organization and interactions in tissues, provide additional layers of information. Significant progress is being made in the development of robust computational methods to determine cell types, states and spatial location, including through the possibility to integrate multiple datasets made publicly available (50–52). Together, these advances have transformed the resolution and scale of tissue cell mapping of IMIDs lesions.

In particular, single-cell RNA-sequencing (scRNA-seq) provides an unbiased method to identify cellular subtypes and/or states defined by distinct molecular programs based on the analysis of thousands of genes, which has the potential to reveal scarce cellular processes relevant to IMIDs pathogenicity. Using scRNA-seq, the profiling of peripheral blood mononuclear cells (PBMCs) from SLE patients allowed to identify the expansion of populations of IFN-stimulated genes (ISG)^hi^ monocytes, plasmacytoid dendritic cells (pDCs), plasma cells (PCs), and cytotoxic natural killer (NK) cells in patients with the highest disease activity (53). An elevated IFN signature was also observed in kidney tubular cells of non-responder patients (42, 43). In seropositive RA, scRNA-seq studies revealed the expansion of fibroblast activation protein-α (FAPα)^+^ fibroblasts that comprised subsets of inflammatory FAPα^+^THY1^+^, and destructive FAPα^+^THY1^−^ fibroblasts, respectively localized in the synovial sub-lining and lining layers (54, 55). Building on these findings, further scRNA-seq studies identified central roles of NOTCH3 signaling in mediating the expansion of THY1^+^ inflammatory fibroblasts (56). Using cytometry by time-of-flight (CyTOF), the same group also described a population of CD4^+^ T peripheral helper (T_PH_) cells organizing B-cell responses and antibody production within pathologically inflamed synovial tissues (57). Through scRNA-seq profiling studies, we and others later identified the contribution of similar T_PH_ cells to inflammatory processes involved in ulcerative colitis and type 1 autoimmune hepatitis respectively (58, 59). In atopic dermatitis (AD) and psoriasis, scRNA-seq analyses of skin immune cells suggested pro-inflammatory macrophage subsets could reactivate programs at play during fetal development to promote angiogenesis and leukocyte infiltration (60). In turn, the study of synovial tissue macrophages identified unique molecular states involved in inflammation resolution and tissue repair, which, when present in low proportions during remission phases, predicted increased risk of RA flares (61).

Multimodal measurements provide ways to associate in single cells analyses, several biological features that are essential to the description of cellular states and functions. Combined transcriptome and T-cell receptor (TCR) sequence analyses allow to generate valuable information about T cell clonal fates, dynamics and effector functions. Such approaches revealed the expansion of T cell clones with cytokine signatures relevant to type 2- and type 3-mediated pathogenicity in lesions of AD and psoriasis respectively (60). In Crohn’s disease (CD), Graham and colleagues recently showed that T cell responses to an immunodominant epitope conserved across gut commensal *Bacteroidales* shifted their cytokine responses from the production of immunoregulatory IL-10 toward IL-17 during disease flares (62). The combined profiling of gene expression and genome-wide chromatin accessibility in single stromal cells suggested the involvement of AP-1 family members in regulating the transition of a discrete subset of LGR5^+^-scleroderma-associated fibroblasts (ScAFs) from healthy to pathological states in the skin of patients with autoimmune systemic sclerosis (SSc) (44). Interestingly, contrary to immune alterations, which were limited to patients with diffuse SSc, ScAFs transitions were universal to both limited and diffuse phenotypes. By unraveling a previously unappreciated profibrotic cellular state, this study offers new opportunities to target fibrosis not only in all forms of SSc but also in other IMIDs with fibrotic complications such as RA and CD.

Disease-associated cell subtypes do not act in isolation but involve cellular interactions between distinct immune and non-immune cell populations, which together participate in shaping the organization of pathophysiological responses in lesions. Profiling cellular interactions is only possible using technologies that provide single cell resolution at scale. We and others recently identified cellular interactions that correlated with disease course (58, 63–69). Using single cell profiling of advanced ileal CD (iCD) lesions, we described a cellular module consisting of 5 different cell populations that accumulated in inflamed lesions of a subset of patients. We also identified the potential drivers of this pathogenic response through the characterization of ligand-receptor pairs preferentially enriched in these patients and confirmed inferred cellular interactions using spatial mapping by multiplex imaging (63, 70). Finally, we developed an analytical strategy allowing to test in large cohorts of patients, the association between the presence of this cellular response in inflamed tissues and relevant clinical parameters. Using this approach, we characterized the cellular and molecular framework in which lies anti-TNF resistance in iCD. As discussed further below, strategies as ours demonstrate the possibility to use scRNA-seq as a way to agnostically identify clinically meaningful molecular layers of patient stratification in polygenic IMIDs, which can serve as a rational basis to implement the design of clinical trials.

### Unraveling immunopathology heterogeneity using single-cell profiling of disease lesions

**Figure 1:**
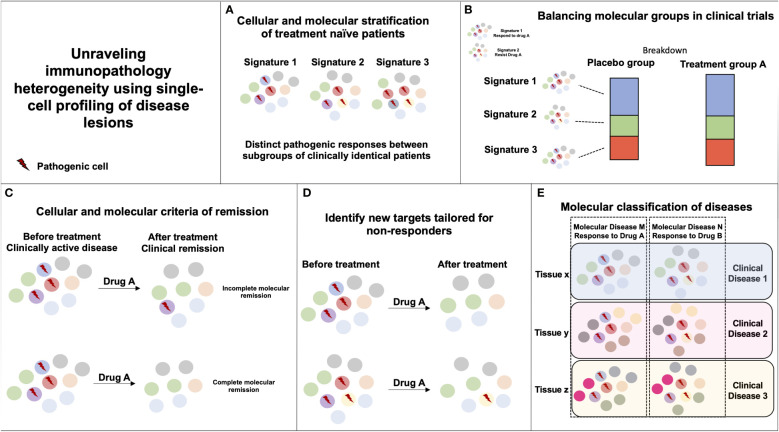
Unraveling immunopathology heterogeneity using single-cell profiling of disease lesions. **(A)** The characterization of distinct immunopathogenic responses in lesional tissues between subgroups of clinically similar patients can help derive stratifying cellular and molecular signatures. **(B)** If two pathogenic responses are causing a similar clinical disease but only signature 1 is responding to Drug A, random or biased enrichment of signature 1 in Placebo vs. Drug A group could mislead to Drug A inefficacy in the disease. **(C)** Monitoring cellular and molecular responses in tissues during the course of treatment can help define molecular endpoints of remission in patients with clinically inactive disease. **(D)** Monitoring cellular and molecular responses in tissues during the course of treatment can help identify immune pathways unaffected by the drug that could be targeted by another therapy. **(E)** Similar immunopathogenic responses occurring in distinct tissues and diseases can be targeted by the same immunotherapy.

The unique potential for single-cell profiling strategies to identify distinct cellular and molecular organization among clinically and histologically homogenous patients provides enormous opportunities to transform clinical care (**Figure 1**). The single-cell profiling of human lesions should help reveal distinct cellular and molecular patterns of disease lesions and stratify patients beyond the clinical and histological diagnosis. It is important, however, that such profiling be done first on lesions naïve of treatment to reduce the confounding variable induced upon exposure to drug and maximize the chances to identify relevant molecular drivers of diseases. In clinical trial design, the identification of disease molecular patterns will enable a balanced distribution of patients with distinct immunopathogenic signatures and help reveal the efficacy of drug treatment in specific subset of patients. Such studies will also help the identification of biomarkers of response to treatment and help maximize therapeutic management. In addition, longitudinal mapping of cellular distributions and their molecular dynamics in lesional tissues during treatment should help identify molecular criteria of therapeutic response that are critically needed, as pathogenic processes in tissues can remain active despite apparent clinical recovery. Quantification of cellular and molecular responses could then serve as primary endpoints to guide therapeutic strategies. Dynamic mapping could also help unravel cellular and molecular processes enriched in responder and non-responder patients. Such results should guide ongoing R&D efforts to identify novel potential targets and combination therapies that associate synergistic drugs targeting distinct molecular pathways. Finally, systematic single-cell profiling of human lesions may reveal pathogenic pathways that are shared among distinct clinical and histological entities, and ultimately lead to a molecular classification of diseases and the design of novel therapies that target molecular drivers of diseases irrespective of the clinical diagnosis (71).

### Clinical translation of single-cell based models to large clinical cohorts

**Figure 2:**
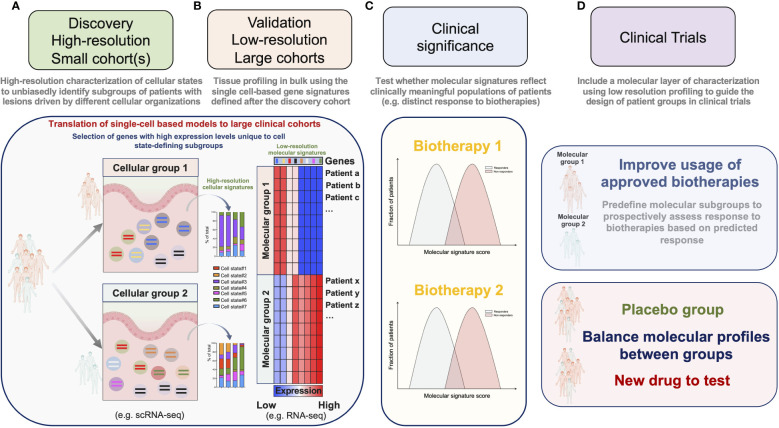
A 2-steps strategy for the implementation of molecular stratification criteria into the design of clinical trials. **(A)** In a “molecular discovery” phase, relevant cellular signatures are first identified by applying high-resolution single-cell profiling to a small cohort of patients. **(B)** In a “clinical association” phase high-resolution-based signatures are confirmed on a large number of patients by projecting the data obtained after low-resolution profiling onto a model devised during the “molecular discovery” phase. **(C)** Molecular scores derived from the agnostically identified signatures are then tested for their association with relevant clinical parameters. **(D)** Molecular criteria are implemented to guide the design of clinical trials.

A major goal of single-cell profiling and multiplex imaging technologies is to associate clinically relevant outcomes with cellular signatures. scRNA-seq enables to agnostically resolve the organization of complex cellular signatures in tissue lesions. Cost and labor constraints, however, restrain its application to small cohorts of patients. Because the distributions of clinical and/or demographic parameters can be highly intercorrelated, it is necessary to disentangle confounding factors in order to extract clinically meaningful molecular signatures, which is only possible with large cohorts. We suggest to overcome these limitations using a 2-steps strategy. In a “molecular discovery” phase, relevant cellular signatures are first identified by applying high-resolution single-cell profiling to a small cohort of patients thoroughly selected by experienced clinicians (**Figure 2A**). In a “clinical association” phase, large numbers of patients are profiled using approaches with mitigated cellular or molecular resolutions. The clinical value of high-resolution-based molecular signatures is then tested by applying a computational method tailored to project the data from samples profiled at lower-resolution onto the model devised during the “molecular discovery” phase (**Figures 2B, C**). This 2-steps strategy hence allows to link at minimal cost relevant metadata (clinical records, genetic information etc.) from a large number of patients with the cellular signatures detected by scRNA-seq.

It is possible to decrease the molecular resolution of scRNA-seq by reducing the number of readouts per cell and/or by reducing the number of single-cells per sample. Besides, the cost of scRNA-seq has largely decreased over the past decade, and is expected to drop even more in the near future (72). These approaches nevertheless require cost and labor-intensive steps of tissue dissociation for single-cell processing and library-preparation, which are expected to remain significant and challenging for implementation in clinical trials. An alternative approach that we and others privileged (63, 64, 68) was to use data from tissues sequenced in bulk for the “clinical association” phase (**Figures 2B, C**). By projecting the data obtained after profiling ileal biopsies with low resolution RNA-seq onto a model derived by the high-resolution scRNA-seq analysis of a small cohort of CD patients, we confirmed our findings on a large number of patients and were able to associate the enrichment of this cellular signature with a poor response to anti-TNF Abs (63). The strength and applicability of RNA seq-based stratification to guide group design in clinical trials were recently demonstrated by Pitzalis and colleagues in RA. In this study, the authors showed that the molecular stratification of RA synovial tissue from patients with inadequate response to anti-TNF Abs outcompeted histopathological classification to define subgroups matching clinical responses. Patients with a low or absent B-cell lineage expression signature in synovial tissue better responded to the IL-6 receptor antagonist tocilizumab, than to B-cell depleting rituximab (73). Ancillary analyses in a follow-up study further revealed that humoral immune response gene signatures associated with response to rituximab and tocilizumab, while patients with a stromal/fibroblast signature were refractory to all medications (41). Overall, these studies thus emphasize the importance of including molecular signatures into clinical algorithms to improve the usage of available biotherapies and guide drug development priorities for refractory patients. We believe that the power of such molecular signatures could be significantly enhanced by applying our 2-steps strategy combining profiling approaches and should be translatable to the design of next-generation clinical trials (**Figure 2D**).

## Conclusion

In this perspective we highlight the enormous potential of single-cell profiling strategies to identify cellular and molecular patterns of diseases within clinically and histologically homogenous cohorts of patients. We reflect on our poor understanding of human disease pathophysiology which stems from limited accessibility of human tissues. We argue that single-cell strategies are uniquely able to provide the granularity required to mine complex disease processes and identify cellular and molecular patterns of diseases that can dramatically enhance knowledge of human diseases pathophysiology and potential therapeutic strategies.

## Data availability statement

The original contributions presented in the study are included in the article/supplementary material. Further inquiries can be directed to the corresponding author.

## Author contributions

All authors listed have made a substantial, direct, and intellectual contribution to the work and approved it for publication.

## Acknowledgments

The authors warmfully thank Pr Regis Josien (CR2TI, Nantes Université) for his critical reading of the manuscript and suggestions. JM is supported by NExT “Junior Talent”, ANR JCJC (ANR-20-CE17-0009) and ARC Programmes labellisés (PGA). NC is supported by a Fondation pour la recherche médicale (FRM) PhD-fellowship (FDM202106013475).

## Conflict of interest

The authors declare that the research was conducted in the absence of any commercial or financial relationships that could be construed as a potential conflict of interest.

## Publisher’s note

All claims expressed in this article are solely those of the authors and do not necessarily represent those of their affiliated organizations, or those of the publisher, the editors and the reviewers. Any product that may be evaluated in this article, or claim that may be made by its manufacturer, is not guaranteed or endorsed by the publisher.
